# Availability of Using Honeybees as Bioindicators of Pesticide Exposure in the Vicinity of Agricultural Environments in Taiwan

**DOI:** 10.3390/toxics11080703

**Published:** 2023-08-15

**Authors:** Chien-Che Hung, Lih-Ming Yiin

**Affiliations:** Department of Public Health, Tzu Chi University, 701, Sec. 3, Zhongyang Road, Hualien City 970374, Taiwan; gavink23@gms.tcu.edu.tw

**Keywords:** bee, bioindicator, exposure, fungicide, herbicide, honey, insecticide, pesticide, dust

## Abstract

While pollinating, honeybees are subject to exposure to a variety of pesticides; with their characteristics of certain foraging distances, they could serve as bioindicators of pesticide exposure in a neighborhood. We conducted a study to assess availability by collecting and analyzing bee samples from 15 apiaries located in East Taiwan and dust samples from the adjacent environment, and by finding relations between both samples. Seventeen pesticides were selected for the analysis using gas or liquid chromatography coupled with mass spectrometry, and eight (three insecticides, two herbicides, and three fungicides) were more frequently detected from bee or dust samples; the levels of these pesticides were mostly under 1000 ng/g. Significant correlation results (*r* ≅ 0.8) between residue concentrations in bees and in dust suggest that honeybees could be a good bioindicator for exposure to herbicides and fungicides within certain ranges. The pesticide contents of sick/dead bees were much higher than those of healthy counterparts regarding any pesticide type, with the mean total concentrations of 635 ng/g and 176 ng/g, respectively. We conclude that honeybees could be used as bioindicators of pesticide exposure; sick/dead bees could serve as a warning sign of the severity of pesticide pollution.

## 1. Introduction

Pesticides are a large group of chemicals or biological agents that control pests for agricultural, gardening, and environmental purposes. Because of the impact of climate change on insect pest breeding, doses and varieties of pesticides are increasingly applied in the present time [1,2], and thus the overuse of pesticides becomes a crucial problem for human and environmental health [3,4]. Many recent studies have confirmed that pesticides applied in agricultural areas may drift over to nearby residential areas [5,6,7,8] and even cause a health concern [9]. In those studies, measuring pesticide residues in dust collected from residential homes adjacent to the agricultural areas was commonly practiced to provide exposure information at the receptor sites; the assessment of potential pesticide exposure from the sources (i.e., agricultural areas with pesticide application), however, needs a sampling approach that gathers information more accurately than house-dust sampling does.

A bioindicator is an organism carrying certain indicative information that is helpful to evaluate the health of an environment or ecosystem. It can be any species, including algae, fungi, plants, animals (sentinel species), and insects, whose population, status, or function (physiological or biological) represents the qualitative status of the environment [10,11]. For the assessment of exposure to pesticides originating from agricultural activities, honeybees (*Apis mellifera*) are considered as an available bioindicator, because they possess various sensitivities to pesticides and the characteristics of limited foraging ranges and little behavioral change due to seasonal changes [12]. There is a history in which honeybees have been used for the biomonitoring of environmental pollutants, including pesticides, heavy metals, radionuclides, and others [13]. An example showing the sensitivity of honeybees to pesticides is Colony Collapse Disorder (CCD) that has been occurring for more than a decade. CCD caused a majority of worker bees to disappear [14], and was later confirmed to be partially attributed to exposure to neonicotinoids, a relatively new type of insecticides [15,16,17]. With the foraging nature that likely translates to contact with pesticides, honeybees used as bioindicators could provide useful information on pesticide exposure caused with agricultural activities. In addition, richness for sampling and convenience for hive mobilization, as desired, make farm-raised honeybees an excellent choice over other wild pollinators for the role of bioindicator.

There have been a number of studies investigating the relationships between bees and pesticides. It was found that insecticides are a lethal threat, whereas herbicides and fungicides could cause a variety of health effects on bees; synergistic effects are even observed when mixtures of various pesticides are applied [18,19,20,21,22,23,24]. Other studies demonstrate significant pesticide exposure for bees, indicating the prevalent use of pesticides, even though several bees’ main activity regions are not intended for pesticide use [25,26,27]. Another study using a geographic information system indicates a strong positive correlation between glyphosate residue concentrations in honey and agricultural land use, suggesting that bee products could be used as indicators for pesticide exposure [28]. Since honey is produced by honeybees, the pesticide residue in honey supposedly originates from bees, especially worker bees; thus, honeybees should work as bioindicators of pesticide exposure as functionally as honey does.

Pesticide use in Taiwan has been one of the highest in the world for recent years [29]. Most people in Taiwan are subject to pesticide exposure because of no significant boundaries between residential and agricultural areas; therefore, an indicator of pesticide exposure around the living places is very much needed. Although honeybees have been used for biomonitoring for years, little is carried out for verifying whether they can be used for exposure assessment. This study is to evaluate the availability of using honeybees as bioindicators of pesticide exposure in the neighborhood of agricultural areas in Taiwan. We expected this approach to derive information from honeybees to help construct the exposure model for communities around the agricultural areas.

## 2. Materials and Methods

### 2.1. Participating Apiaries and Sample Collection

**Figure 1:**
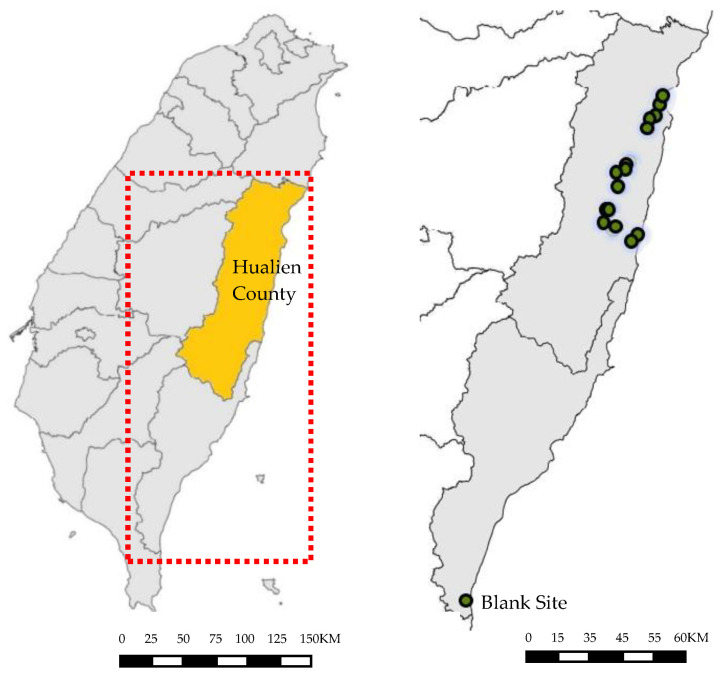
Location of apiaries in East Taiwan.

This study was conducted in Hualien County, a rural area located in East Taiwan, from October 2020 to October 2022. Because of the sub-tropic and tropic climate in the region, the apiaries operated all-year-round regardless of seasons. There were 15 apiary teams within the area, which were willing to collaborate with this study and allowed the study team to take necessary samples during the honey collecting periods. These apiaries were adjacent to fruit gardens and/or betel nut groves; 1 of the 15 apiaries had a site in an experimental forestry station to the south of Hualien, which was used as a blank because of no pesticide applications (Figure 1).

At each apiary, we collected worker bee and honey samples from half of the hive boxes. Healthy and sick/dead bees inside and outside the hive boxes were collected in 50 mL centrifuge tubes using a pair of Teflon^®^ tweezers, respectively. Approximately 15~20 bees of each type (i.e., healthy and sick/dead) were placed in a centrifuge tube and analyzed as a composite sample. Honey was collected and stored in a 50 mL centrifuge tube using a Teflon^®^ spoon or a disposable dropper. Bee samples were taken as planned, whereas honey was collected upon availability. On average, 3~5 composite samples of bees (healthy or sick/dead) were collected from each apiary. Environmental dust was collected with a changeable-bag vacuum system (Makita DVC261ZX18, Makita Co., Aichi, Japan) up to 10~15 g from public places in the neighborhood around an apiary within a radius of 300, 1000, or 5000 m. Prior to the analysis, dust samples were transferred into brown glass bottles for storage at room temperature with relative humidity controlled below 60%, while bee and honey samples were temporarily stored in a 4 °C portable refrigerator on site and transferred to an ultra-low temperature freezer at −80 °C in a laboratory.

### 2.2. Sample Treatment and Analysis

We selected a range of pesticides that were known to be commonly sold in Taiwan to be the analytes of interest. They were acetamiprid, carbaryl, carbofuran, chlorpyrifos, cypermethrin, fipronil, imidacloprid, permethrin, prallethrin, and tetramethrin for insecticides, glufosinate, glyphosate, and paraquat for herbicides, and benomyl, chlorothalonil, mancozeb, and propineb for fungicides. We followed and modified analytic methods that were previously published for sample treatment and an analysis using gas-chromatography–mass-spectrometry (GC-MS, Agilent 6890/5973, Agilent Technologies, Santa Clara, CA, USA) or liquid-chromatography–mass-spectrometry (LC-MS, Agilent 1200/6460A, Agilent Technologies, Santa Clara, CA, USA) [30,31]. The information of target pesticides of this study is listed in Table 1.

Bee samples were first weighed, homogenized, and added to 50 mL of ethyl acetate for 30 min ultrasonic extraction; the mixture was then centrifuged at 5000 rpm for 10 min. The supernatant was concentrated, reconstituted with 500 μL of n-hexane, filtered, and transferred to an insert vial for the GC-MS or LC-MS analysis. For dust sample treatment, three grams of dust from each sample was first weighed, and the remaining procedures were similar to the treatment of bee samples, which were processes of extraction, concentration, reconstitution with n-hexane, filtration, and the analysis using GC-MS or LC-MS. Honey samples were diluted with deionized water with the ratio of 1:0.3~1 (honey/water), depending on the viscosity. The diluted sample solutions were introduced to C18 cartridges (Strata C18 SPE cartridge, Phenomenex, Torrance, CA, USA) with pressure for solid-phase extraction. The cartridges were washed with deionized water and dried with flushing nitrogen; the eluate was then eluted with n-hexane, and processed for the chromatographic analysis after concentration and solvent reconstitution.

The GC-MS system used in this study was the same as reported previously [5], with different settings regarding the flow rate, split mode, and temperature profile. Ultra-purity helium (99.9995%) was used as a carrier gas with the constant flow rate set at 1.0 mL/min; the inlet condition was set at 280 °C with a split model (30:1). The oven temperature began at 60 °C, and was increased to 160 °C at a rate of 20 °C/min, to 300 °C at 10 °C/min, and held at 300 °C for 8 min (total runtime: 27 min). The LC-MS system was equipped with a C18 column (Agilent Poroshell 120 EC-C18, 3.0 × 100 mm, 2.7 μm, Agilent Technologies, Santa Clara, CA, USA), and deionized water and methanol, with both containing 10 mM of ammonium acetate, were used for gradient separation. Twenty percent methanol was used for elution in the beginning, and the percentage increased linearly to 50% at 10 min, to 70% at 13.5 min, to 71% at 20 min, and to 100% at 29 min; the elution continued with 100% methanol until 35 min. The limits of detection (LODs) for all analytes using GC-MS or LC-MS were determined to be 1 ng/g or lower. The recovery rates were between 94% and 99% by spiking samples with standards; the coefficients of variance of the overall analysis were lower than 5%.

### 2.3. Data Management and Statistical Analysis

The correlation analysis was used to examine the association between pesticide concentrations in bee samples and those in environmental dust samples. Because not every pesticide in a bee or dust sample was detectable, we summarized the detected concentrations by pesticide type (i.e., insecticide, herbicide, and fungicide) for an individual sample, and used them for the correlation analysis. The concentration of each sample that was undetectable was replaced by half the LOD. A general linear model was employed for the two-way analysis of variance (ANOVA) to examine the difference in pesticide residue in healthy and sick/dead bees, and whether the distribution patterns of insecticides, herbicides, and fungicides were different between these two types of bees. All statistical analyses were performed using the SPSS statistical software package version 23.0 (SPSS Inc., Chicago, IL, USA, 2015).

## 3. Results

We collected a total of 112 composite samples of bees (healthy, 52; sick/dead, 60), 28 honey samples, and 180 environmental dust samples. Among the 17 target pesticides, 8 were frequently detected from bee or dust samples, whereas nearly nothing was detected from honey (Table 2). Bee or dust samples collected from the blank site were all under LODs, supporting the quality control of the sample analysis. The detection rates of insecticides (>40%) or fungicides (>20%) for bees and dust were similar, except those of herbicides were low for bee samples (<33%) and high for dust samples (>52%). Among the types of pesticides, data from bee and dust samples indicate that fungicides were less abundant than the others in this study (i.e., lower detection rates and median concentrations). For the comparison of pesticide concentrations, it appears that herbicides and fungicides were richer in dust than in bees, whereas insecticides did not quite follow this pattern. Most of the pesticide levels were under 1000 ng/g, except the maximum values of certain pesticides in dust.

To evaluate the availability of using honeybees as bioindicators, we conducted a correlation analysis between pesticide concentrations in bees and those in environmental dust collected from various distances surrounding the apiaries (Table 3). As mentioned previously, the sums of pesticide concentrations of the same types were used in the analysis to avoid skewed results due to non-detects. All except correlation for insecticides in the near or middle range were significant (*p* < 0.05), indicating the associations between pesticide residue concentrations in honeybees and in dust of the nearby environment, especially those for herbicides and fungicides within the near and middle ranges (*r* ≅ 0.8). All three types of pesticides yielded significant correlation results in the far range, albeit merely fair, suggesting that the foraging distances of honeybees could reach up to 5000 m. The correlation data further confirm that using honeybees as bioindicators is feasible.

**Figure 2:**
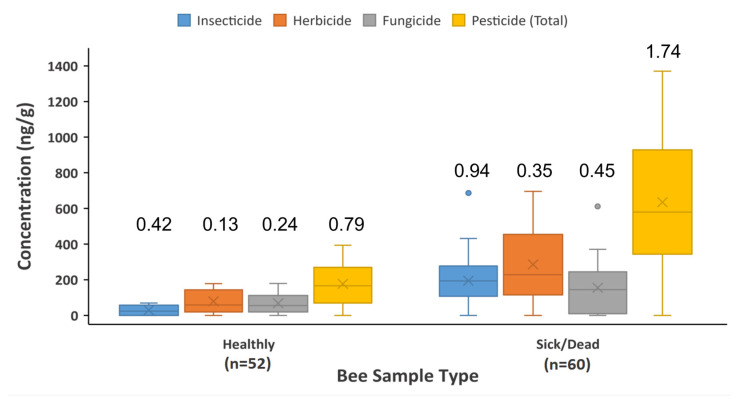
Pesticide detection and levels of healthy and sick/dead bees. (The number above each box denotes the mean number of detected pesticides per bee. The line and “X” in the box depict median and mean values, respectively).

We further analyzed the data of pesticides in bees with the health status, and found significant differences in the number of total detected pesticides per bee and total pesticide concentration between healthy and sick/dead bees (0.79 pesticides/bee and 176 ng/g, and 1.74 pesticides/bee and 635 ng/g, respectively; *p* < 0.001) (Figure 2). The average number of detected pesticides of each type per bee is at least two times higher for sick/dead bees than healthy counterparts, except that of fungicide is 1.9 times higher (0.45/0.24). Similarly, the mean concentration of each pesticide type is higher in sick/dead bees than healthy ones, but the differences among the types are not quite similar. The levels of insecticides and herbicides of sick/dead bees are at least 3.6 times higher than those of healthy ones, whereas the concentration difference between healthy and sick/dead bees for fungicides is merely 2.2-fold. Given the abovementioned data, it appears that sick/dead bees might have been exposed to much more insecticides and herbicides than fungicides. The ANOVA model further confirmed the difference between pesticide distribution patterns of healthy and sick/dead bees by showing a significance level of 0.001.

## 4. Discussion

Compared with previous studies [25,26], the detection rates and concentrations of pesticides in bees in this study were relatively high; the mean detection rate was roughly 31%, and the median concentrations of insecticides and herbicides were above 180 ng/g (Table 2). One study that investigated pesticide exposure for wild bees and butterflies in the cultivated agricultural areas located in mid-northern Missouri, USA found that most of the total pesticide concentrations in bees were under 100 ng/g, with the average detection rate being around 25% [25]. Another study attempted to find out whether border plantings could be pesticide sources for bees, and they detected several common pesticides from over 25% of honeybee samples with the median concentration of each pesticide type being around 100 ng/g [26]. The reason that this study yielded relatively high pesticide levels could be that the inclusion of sick/dead bees elevated the overall means (Figure 2). For those two previous studies, bee samples were collected on site with certain nets or traps, and thus those bees were presumably healthy. As for our healthy bees’ data (Figure 2), the levels were relatively low and seemingly of no substantial difference from those of other studies, despite exposure to different types of insecticides, herbicides, and fungicides. Thus, in terms of healthy bee sampling, the pesticide exposure found in this study was not particularly higher than that reported by others; with the inclusion of sick/dead bees, however, the exposure profile became different. Although the quantity of sick/dead bees might be relatively small, they represented the high end of pesticide exposure, which was valuable and necessary for a complete assessment of pesticide exposure. If a significant number of dead/sick bees is observed with hive boxes, that may indicate ongoing pesticide pollution, to which attention should be paid.

Sick/dead bees resulted in not only an increase in pesticide concentration, but also an enhancement in detection rates, which was beneficial to the quantitative analysis. It is of no surprise to see higher insecticide levels in sick/dead bees than in healthy bees (~6 times), because insecticides are lethal and definitely a cause of death. The levels of herbicides and fungicides are also elevated and could be the causes of sickness, considering that both types of pesticides are less toxic than insecticides. Given the fact that the mean concentration of herbicides was much higher than that of fungicides in sick/dead bees (286 ng/g vs. 155 ng/g, Figure 2), the sick bees could be mostly attributed to exposure to herbicides. It is also likely that the death and sickness of bees could have been caused by the synergistic effect of mixtures of a variety of pesticides, with 1.74 pesticides detected from a sick/dead bee on average [23,24].

The correlations between herbicides/fungicides in bees and in environmental dust were significantly strong (r ≅ 0.8) within the near and middle ranges (<1000 m), suggesting that bees could be used as bioindicators of exposure to herbicides or fungicides in a vicinity as a result of pesticide drift. Of a certain surprise, no significant correlation was, however, found for insecticides within the same ranges. One of the reasons may have been the toxicity of pesticides of different types. As mentioned in the previous paragraph, insecticides are lethal and probably acute, and herbicides and fungicides are less toxic and chronic; thus, there must have been a certain number of honeybees dying outside the apiary after exposure to insecticides, which would not be accounted for in samples. Honeybees that were exposed to herbicides and/or fungicides could return to an apiary and serve as samples. Consequently, the strong correlations for herbicides and fungicides may have resulted from sufficient bee samples, while the poor correlations for insecticides may have resulted from insufficient bee samples.

The detection rates for honey samples were relatively low, which were 9 detects out of 224 (8 pesticides × 28 samples); in addition, the maximum values were far lower than those of bee or dust samples. Similarly, low levels of pesticides in honey were reported in previous studies [32,33], which analyzed bee products for fungicides and always found lower residue concentrations in honey than in pollen. The reason for low residue concentrations in honey is likely due to detoxification with enzymes. As honey is produced with a series of a worker bee’s tasks, including flower nectar suction and regurgitation among bees, detoxifying enzymes inside the bodies (e.g., 450 s), albeit relatively few [34,35], could metabolize pesticides in the nectar (later turned into honey) to a certain extent. A similar metabolism of pesticides may be driven by microbes in the bee gut. The majority of the honey samples under detection in this study are a good sign in the perspective of food safety, but few detects may still be of concern for approaching or exceeding the maximum residue levels.

It appears that herbicides are prevalently used in modern agriculture, as shown with the bee and dust data. Paraquat, one of the detected herbicides, has been banned for use in Taiwan since February 2020, but is still detected from bee and dust samples. The degradation mechanisms of paraquat are considered to be slow [36], but the water solubility is high [37], suggesting that paraquat would not degrade soon but could be easily washed out with rain. Therefore, paraquat detected in this study should more likely originate from recent applications after the ban than from remaining residues of paraquat use in the past. Source management for paraquat is recommended for an effective control.

There are limitations in the study. First of all, we observed and used dead/sick bees as samples, but had difficulty knowing about their percentage of the whole bee population. Had we had this information, the weighting of healthy bees’ and sick/dead bees’ data with that should have helped construct a fit model of pesticide exposure for bees. This is the information that we can strive to collect in future work. Secondly, we may have lost honeybee samples to any possible ongoing CCD, of which we were never aware. Given the fact that imidacloprid is the major CCD affecting insecticides [38] and our result of the detection of imidacloprid from samples is rare, this limitation is considered minimal. Thirdly, dust sampling was subject to location accessibility (e.g., private lands), and the sample collection could not be as complete as bee sample collection; thus, such a reduction in sample size could lead to low statistical significance. Fortunately, most of the correlation results are significant, meeting the goal of the study. Speaking of limited accessibility to certain areas, honeybees, once confirmed to serve in the role of bioindicator, could be useful to overcome problems of limited access to certain sites.

## 5. Conclusions

We conclude that honeybees could be used as bioindicators of exposure to pesticides in an adjacent environment, especially herbicides and fungicides. Honey, as a bee product, was not suitable for use as a bioindicator, because of the low detection rates. Sick/dead bees contained more contents of insecticides, herbicides, and fungicides than healthy bees did, suggesting that an observation of a significant number of sick/dead bees could be a warning sign of the severity of pesticide pollution.

## Figures and Tables

**Table 1:** General information of target pesticides.

Type and Name	Analytical Method	Retention Time (min)	Main Use for Agriculture, Environmental Sanitation, or Both
**Insecticide**			
Acetamiprid	LC-MS	2.31	Agriculture
Carbaryl	GC-MS	27.21	Agriculture
Carbofuran	GC-MS	23.92	Agriculture
Chlorpyrifos	GC-MS	8.62	Both
Cypermethrin	GC-MS	22.71	Agriculture
Fipronil	GC-MS	6.31	Both
Imidacloprid	LC-MS	1.93	Both
Permethrin	GC-MS	21.92	Both
Prallethrin	GC-MS	21.43	Environmental sanitation
Tetramethrin	GC-MS	20.51	Environmental sanitation
**Herbicide**			
Glufosinate	LC-MS	1.39	Agriculture
Glyphosate	LC-MS	1.13	Agriculture
Paraquat	LC-MS	3.29	Agriculture
**Fungicide**			
Benomyl	LC-MS	5.41	Agriculture
Chlorothalonil	LC-MS	7.43	Agriculture
Mancozeb	GC-MS	11.76	Agriculture
Propineb	GC-MS	12.44	Agriculture

**Table 2:** Pesticide concentrations by type in different sample media.

Sample Type	Pesticide	DF (%)	50th Percentile (ng/g)	75th Percentile (ng/g)	Maximum (ng/g)
**Bee** **(n = 112)**	**Insecticide**				
	Carbaryl	50.30	114.18	126.25	726.58
	Carbofuran	44.68	207.63	254.31	823.22
	Chlorpyrifos	41.03	366.21	403.64	331.35
	**Herbicide**				
	Glyphosate	32.07	231.26	247.11	523.26
	Paraquat	16.11	184.32	246.31	677.85
	**Fungicide**				
	Benomyl	21.58	71.19	73.39	763.09
	Mancozeb	22.19	126.76	215.33	449.11
	Propineb	25.08	78.86	105.22	892.26
**Honey** **(n = 28)**	**Insecticide**				
	Carbaryl	3.57	LOD	LOD	50.23
	Carbofuran	ND	LOD	LOD	LOD
	Chlorpyrifos	7.14	LOD	LOD	79.22
	**Herbicide**				
	Glyphosate	ND	LOD	LOD	LOD
	Paraquat	ND	LOD	LOD	LOD
	**Fungicide**				
	Benomyl	3.57	LOD	LOD	43.37
	Mancozeb	7.14	LOD	LOD	126.25
	Propineb	10.71	LOD	LOD	79.18
**Dust** **(n = 180)**	**Insecticide**				
	Carbaryl	47.78	337.26	368.54	2314.35
	Carbofuran	59.44	206.54	245.33	2033.54
	Chlorpyrifos	41.11	191.25	197.65	2778.52
	**Herbicide**				
	Glyphosate	62.22	306.54	377.52	2788.5
	Paraquat	52.22	317.85	404.17	1926.3
	**Fungicide**				
	Benomyl	22.78	207.15	236.74	885.39
	Mancozeb	36.11	155.23	283.31	1943.25
	Propineb	23.89	289.65	303.97	7336.47

DF: detection frequency; LOD: limit of detection; ND: not detected.

**Table 3:** Correlation between pesticide concentrations in bees and dust collected at distances.

Pesticide Type	Distance from Apiary to Dust Collecting Location					
	Near (0–1000 m) n = 84		Middle (300–1000 m) n = 53		Far (1000–5000 m) n = 43	
	*r*	*p*	*r*	*p*	*r*	*p*
**Insecticide**	−0.386	0.155	0.075	0.791	0.674	0.006
**Herbicide**	0.838	<0.001	0.811	<0.001	0.467	0.08
**Fungicide**	0.783	<0.001	0.834	<0.001	0.752	<0.001

## Data Availability

The data presented in this study are available on request from the corresponding author.
